# BRM: From Skin-Reducing Mastectomy to the New Concept of Breast Reshaping Mastectomy

**DOI:** 10.3390/jcm14041350

**Published:** 2025-02-18

**Authors:** Valerio Lorenzano, Andrea Vittorio Emanuele Lisa, Valeriano Vinci, Benedetta Agnelli, Alessia Lozito, Marco Klinger, Alessandro Mela, Martina Caruso, Francesco Klinger

**Affiliations:** 1 Scuola di Specializzazione in Chirurgia Plastica, Ricostruttiva ed Estetica, Università Degli Studi di Milano, Via Festa del Perdono 7, 20122 Milan, Italy; valerio.lorenzano@gmail.com (V.L.); alessandromela3@gmail.com (A.M.); 2 Department of Plastic and Reconstructive Surgery, European Institute of Oncology, IRCCS, 20141 Milan, Italy; 3 PhD Program in Applied Medical-Surgical Sciences, Department of Surgical Sciences, University of Rome “Tor Vergata”, Viale Oxford 81, 00133 Rome, Italy; 4 Department of Biomedical Sciences, Humanitas University, Via Rita Levi Montalcini 4, Pieve Emanuele, 20090 Milan, Italy; valeriano.vinci@hunimed.eu (V.V.); benedetta.agnellimd@gmail.com (B.A.); alessialozito@gmail.com (A.L.); martina-caruso@hotmail.it (M.C.); 5 Plastic Surgery Unit, Department of Medical Biotechnology and Translational Medicine BIOMETRA, Humanitas Clinical and Research Hospital, Reconstructive and Aesthetic Plastic Surgery School, University of Milan, Via Manzoni 56, Rozzano, 20090 Milan, Italy; marco.klinger@humanitas.it (M.K.); effeklinger@gmail.com (F.K.)

**Keywords:** skin-reducing mastectomy, conservative mastectomy, breast reshaping mastectomy, gigantomastia, breast hypertrophy, Wise pattern mastectomy

## Abstract

**Background:** Macromastia is a well-known issue in breast reconstruction. Skin-reducing mastectomy (SRM) was introduced as a skin-sparing mastectomy that utilizes a skin reduction pattern similar to breast reduction or breast lift surgery, specifically to manage hypertrophic and pendulous breasts. Over time, numerous authors have contributed to refining the SRM technique, leading to the development of various technical variants. However, the diversity of approaches inspired by SRM has created confusion, and clear surgical indications are lacking. **Methods:** We propose a unifying concept called breast reshaping mastectomy (BRM), which encompasses all techniques based on SRM principles. The BRM aims not only to preserve and reduce the breast skin envelope but also to immediately reshape it for a more aesthetic outcome. This approach is applicable to all mastectomies where skin envelope preservation (with or without the nipple-areola complex) is oncologically safe, a modification of breast skin coverage is needed for better aesthetic results, and an implant-based reconstruction is planned. **Results:** To define the BRM concept, we reviewed the existing literature on SRM and its related techniques. Our analysis focused on four key elements: skin incision pattern, implant coverage strategy, nipple-areola complex (NAC) management, and the choice between two-stage and direct-to-implant reconstruction. **Conclusions:** By integrating these four components into a single surgical framework, BRM provides a structured approach to breast reconstruction that enhances both oncologic safety and aesthetic outcomes. Standardizing these techniques could help clarify surgical indications and improve reconstructive planning for patients undergoing skin-sparing mastectomy.

## 1. Introduction

Conservative mastectomies are now considered a reliable and valid option for managing multicentric disease or for treating local recurrences after breast-conserving treatments [1].

The history of conservative mastectomies began with the introduction of the modified radical mastectomy (MRM) by Madden in 1965 [2]. Toth and Lappert later introduced the concept of the skin-sparing mastectomy (SSM) in 1991 [3,4], which was further developed by Carlson et al. in 1997 [5]. The next advancement was the nipple-sparing mastectomy (NSM), which preserves the nipple–areolar complex during an SSM. This approach is considered safe if the nipple–areolar ducts are confirmed to be free of disease via frozen section examination, intraoperative radiotherapy is performed, or definitive histology and preoperative imaging confirm the nipple areolar complex (NAC) to be free from disease [1].

The latest conservative mastectomy technique to be described is the skin-reducing mastectomy (SRM). Although the term was coined by Nava et al. in 2006, the procedure was first described by Bostwick in 1990 as an esthetic solution for prophylactic mastectomies [6,7]. Hammond et al. also applied this technique to a cohort of patients, with results reported in 2002 [8]. The SRM is a type of skin-sparing mastectomy that employs a skin reduction pattern similar to those used in breast reduction or breast lift surgeries, making it particularly suitable for patients with hypertrophic or pendulous breasts. When autologous reconstruction is contraindicated, the SRM offers clear advantages. It addresses skin redundancy, achieves a desirable breast shape, and provides favorable scar placement that can be harmonized with contralateral breast surgery.

**Figure 1:**
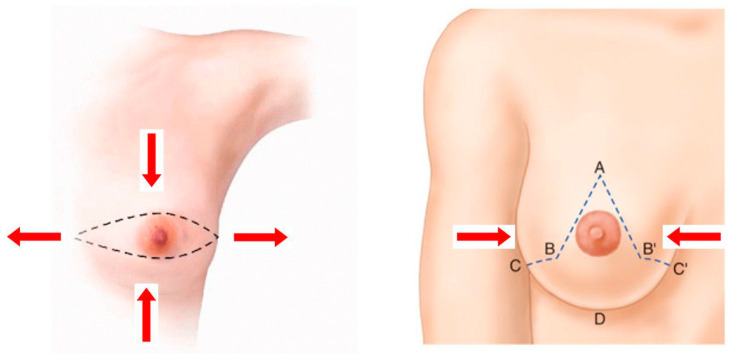
Comparison between Wise pattern skin-reducing mastectomy (**right**) and skin-sparing mastectomy with a Stewart transverse skin incision (**left**). We can see how the incision pattern changes the final shape of the breast after surgery. The transverse skin incision leaves a wide and flat breast; on the contrary, Wise pattern incision can lift, narrow, and project the breast. A = new nipple position. B = upper closure of vertical incision; B and B’ are sutured together. C = lower closure of vertical incision; C and C’ are sutured together. D = infra mammary fold.

Comparing a Wise pattern skin-reducing mastectomy with an SSM using a Stewart transverse skin incision highlights how incision patterns influence the final breast shape. The transverse skin incision results in a wide, flat breast that may not match the contralateral breast in terms of scars and shape [9]. Conversely, the Wise pattern incision can lift, narrow, and project the breast, creating a more symmetrical and esthetically pleasing result (Figure 1) [10,11,12,13].

However, the SRM also presents challenges. The width-to-length ratio of the Wise pattern incision is less favorable for mastectomy flap viability compared to the Stewart transverse incision pattern [14]. Furthermore, tension at the T-junction closure in the SRM is associated with a high risk of skin necrosis, reported in up to 27% of cases [5,8,15,16,17,18,19]. These disadvantages are mitigated by the option to use the adipodermal layer of the lower Wise excision pattern as a pedicled adipodermal flap. This flap can be sutured to the lower edge of the detached pectoralis major muscle, creating a musculodermal pocket that reduces tension on the overlying mastectomy flaps, provides a more natural ptotic breast appearance, and completely covers the implant with vascularized tissue. These innovations align with the original technique described by Nava.

Since its introduction, numerous authors have contributed to refining the SRM technique, resulting in a variety of technical modifications.

In our opinion, the multitude of techniques derived from the philosophy of the SRM should be unified into a single surgical method. This method should prioritize not only sparing and reducing the breast skin envelope but also reshaping the breast for improved esthetic outcomes. We propose the term breast reshaping mastectomy (BRM) to describe this concept. The BRM can be applied to all mastectomies where skin envelope preservation (with or without the NAC) is oncologically safe, where reshaping the skin cover is necessary to achieve an esthetic result, and where implant-based reconstruction is planned.

We identified four key components of a breast reshaping mastectomy:

1. Skin incision pattern.
2. Implant coverage strategy.
3. NAC strategy.
4. Two-stage vs. direct-to-implant reconstruction technique.

These components offer various options that can be combined to create a tailored surgical approach. This variability complicates the analysis of the impact of individual choices within a single technique.

**Figure 2:**
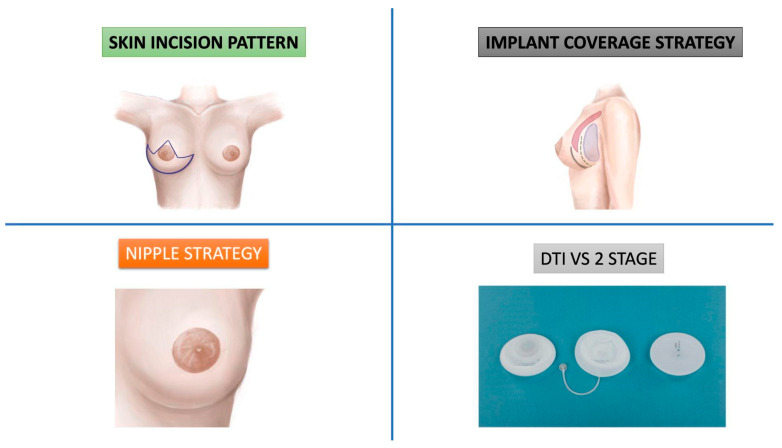
The four items that constitute the concept of the BRM: skin incision pattern, implant coverage strategy, NAC strategy, two-stage vs. direct-to-implant technique.

The aim of our study is to review the existing literature on the SRM and related techniques to collect, describe, and analyze the four components that define the concept of the BRM (Figure 2).

## 2. Materials and Methods

A retrospective review of the literature was performed using Medline, PubMed, CINAHL, AMED, EMBASE, Google Scholar, and the Cochrane Library databases to identify relevant articles. The following keywords were used: skin-reducing mastectomy, Wise pattern breast reconstruction, macromastia AND breast reconstruction, Wise pattern mastectomy, skin-reducing mastectomy, dermal flap AND breast reconstruction, dermal flap AND mastectomy. The search included all study designs and was limited to articles published in English up to July 2022. The bibliography of each relevant article was also reviewed for additional papers not identified in the initial search.

### 2.1. Inclusion and Exclusion Criteria

The study included articles dealing with mastectomy, either therapeutic or prophylactic, that provided an adequate description of the reconstructive surgical technique [1,2,3,4,5,6,7,8,9,10,11,12,13,14,15,16,17,18,19,20,21,22,23,24,25,26,27,28,29,30,31,32,33,34,35,36,37,38,39,40,41,42,43,44,45,46,47,48,49,50,51,52,53,54,55,56,57,58,59,60,61,62,63,64,65,66,67,68,69,70,71,72,73]. The selection was based on the presence of four key items relevant to our research: skin incision pattern, two-stage vs. direct-to-implant technique, implant coverage strategy, and NAC strategy. Articles focusing on esthetic breast surgery and autologous breast reconstruction were excluded.

**Figure 3:**
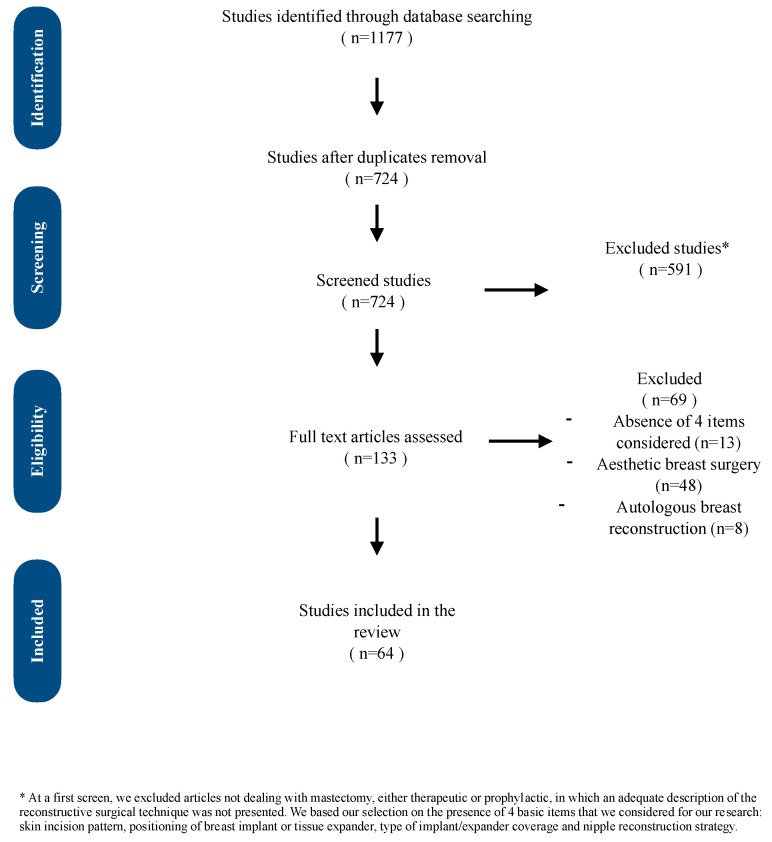
Diagram showing the process of article selection.

From the initial search, 133 titles were identified. Non-English language and duplicate articles were excluded, leaving 64 articles that met the inclusion criteria. These articles were reviewed in detail to form the basis of this study. The article selection process is illustrated in Figure 3.

### 2.2. Data Extraction and Analysis

Data from the selected articles were systematically extracted and organized into predefined categories. These categories included the following:

- **Surgical procedure:** procedure type, indications, proposed technical advantages, markings, skin incision pattern, positioning of breast implant or tissue expander and its related volume, type of implant/expander coverage, dermal flap type, nipple strategy, contralateral breast strategy, and average operating time.
- **Study design:** number of patients, number of breasts, number of breast implants and tissue expanders placed, number of nipple grafts, and average follow-up duration.
- **Patient characteristics:** mean age, BMI, mastectomy weight, history of radiotherapy or chemotherapy, and smoking habits.
- **Surgical complications:** wound dehiscence, complete or partial necrosis of the nipple-areolar complex, delayed wound closure, skin necrosis, implant exposure or loss, seroma, hematoma, capsular contracture, surgical site infection, breast implant infection, and other minor complications.

A descriptive analysis was performed on the collected data to summarize the findings from the literature (Table 1, Table 2 and Table 3). Continuous variables were reported as means or medians (as appropriate), while categorical variables were reported as frequencies or percentages. No formal statistical comparisons or meta-analyses were conducted, as the study aimed to provide a comprehensive review rather than test specific hypotheses.

## 3. Results

**Figure 4:**
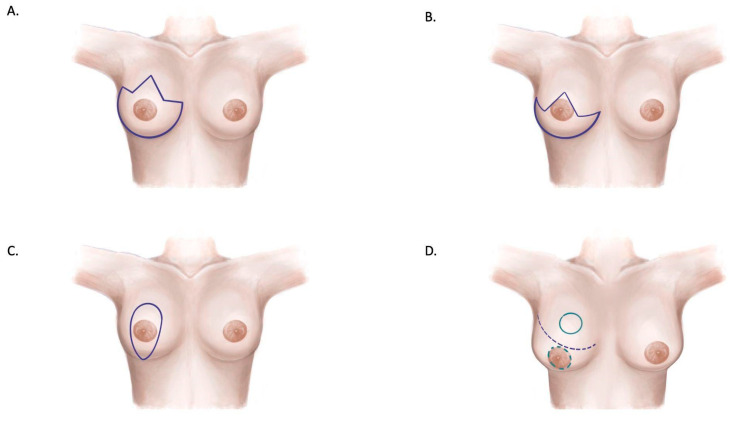
The different types of incision described in the literature for skin-reducing mastectomy procedures. (**A**) Standard Wise pattern. (**B**) Modified Wise pattern. (**C**) Vertical pattern. (**D**) Passot pattern.

**Figure 5:**
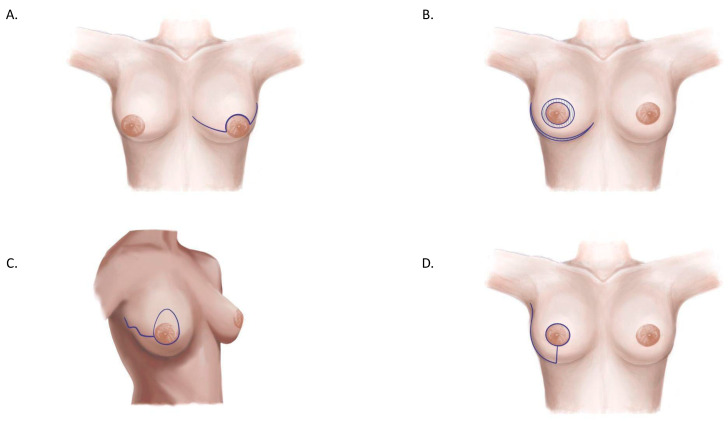
(**A**) Batwing pattern, (**B**) periareolar and inframammary fold incisions, (**C**) raquet pattern, (**D**) B-mammaplasty pattern.

This review identified various skin incision patterns commonly used in breast reconstruction with a mastectomy (Figure 4 and Figure 5). The Wise pattern incision emerged as the most frequently described technique across 34 studies. This approach offers high reshaping potential, making it particularly effective for patients requiring significant contouring. However, its use is associated with a relatively high complication rate, with reports of skin necrosis occurring in up to 27% of cases, as well as delayed wound closure [7,30,31]. A modification of the Wise pattern incision was described in 16 studies. This modified approach aims to reduce flap tension and minimize the risk of necrosis while preserving the reshaping capabilities of the original technique [10,31,33]. The vertical pattern, discussed in four studies, is most suitable for addressing mild to moderate ptosis [3,39,40]. While it has a lower complication rate compared to other methods, it is limited in its ability to manage severe ptosis. The Passot and batwing patterns, which were evaluated in six studies, have shown efficacy in achieving vertical reduction and are associated with lower complication rates. However, these techniques have limited reshaping potential compared to the Wise pattern incision [28,29].

**Figure 6:**
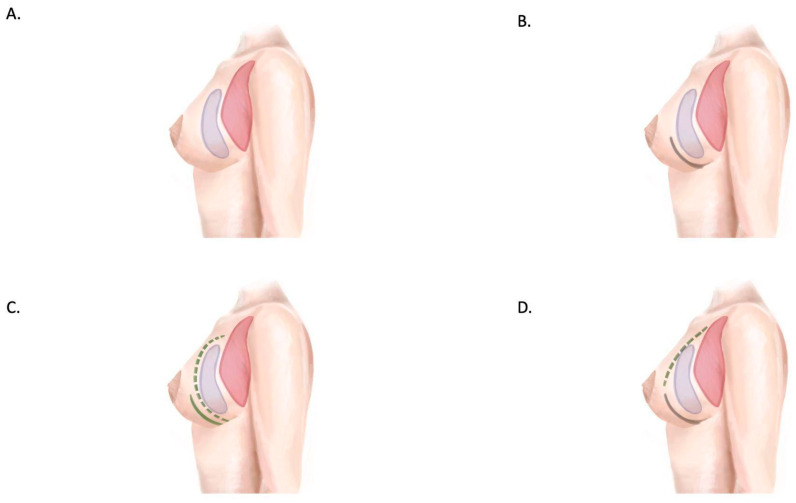
Prepectoral implant coverage strategies. (**A**) Subcutaneous (+/− barrier dermal flaps), (**B**) partial coverage with ADF, (**C**) complete coverage with ADM + ADF overlay, (**D**) complete coverage with ADF + ADM/dermal patch or synthetic mesh. Pectoralis major muscle in red, implant in purple, ADF represented by continuous green line, and ADM/mesh/dermal patch represented by green dotted line.

**Figure 7:**
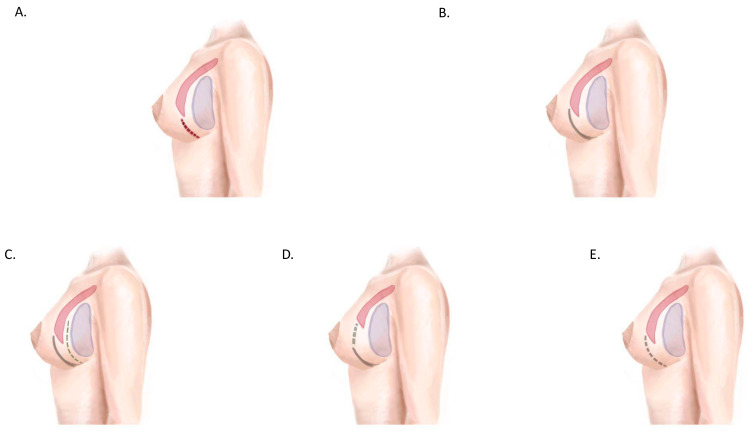
Subpectoral implant coverage strategies. (**A**) Complete muscular/musculo-fascial coverage. (**B**) Complete coverage with musculodermal pocket (composed by pectoralis major muscle superiorly and ADF inferiorly). (**C**) Complete coverage with muscolodermal pocket + ADM/mesh underlying ADF. (**D**) Complete coverage with muscolodermal pocket + ADM interposed between muscle and ADF. (**E**) Complete coverage with muscle superiorly and ADM inferiorly. Pectoralis major muscle in red, implant in purple, ADF represented by continuous green line, and ADM/mesh/dermal patch represented by green dotted line.

Regarding implant coverage strategies, the musculodermal pocket was extensively analyzed in 37 studies (Figure 6 and Figure 7). This approach provides complete vascularized coverage for implants, ensuring adequate protection and support [42,44,50]. However, the technique presents certain challenges related to donor site limitations, including the requirement for sufficient viable tissue and the potential for esthetic and functional impacts at the donor site. Acellular dermal matrix (ADM)-based coverage, particularly in prepectoral reconstruction, has gained popularity due to its effectiveness. [52,53]. Nevertheless, it is associated with higher costs and variable complication rates, requiring careful consideration during surgical planning. Prepectoral placement with synthetic meshes represents an emerging alternative that offers promise [54,56]. Despite its potential, long-term outcomes remain inadequately studied, highlighting the need for further research in this area.

This review also examined strategies for managing the nipple–areola complex (NAC). Immediate NAC preservation, discussed in 22 studies, is associated with superior esthetic outcomes. [46,47]. However, it necessitates careful patient selection to ensure oncological safety and the viability of the skin flap. Auto-free nipple grafting, evaluated in 25 studies, is an effective option in cases where immediate preservation is not feasible [25,51]. This approach provides a reliable solution while accommodating oncological concerns. Conversely, delayed NAC reconstruction offers oncological safety and allows for greater flexibility in planning. However, it comes at the expense of immediate esthetic outcomes, which may affect patient satisfaction in the short term [64].

Finally, the comparison between two-stage and direct-to-implant (DTI) reconstruction revealed notable differences in outcomes. DTI reconstruction was associated with a higher overall complication rate, reported at 30.3%, including a skin flap necrosis rate of 9.7% [65,72]. This approach is best suited for carefully selected patients who present favorable anatomical and oncological profiles. On the other hand, two-stage reconstruction demonstrated a lower overall complication rate of 20.3% and is the preferred choice for patients with compromised flap viability or significant risk factors [34,65]. These findings underscore the importance of individualized surgical planning in breast reconstruction with mastectomy to optimize both safety and esthetic outcomes.

## 4. **Discussion**

Breast reshaping mastectomy (BRM) represents a conceptual advancement in breast surgery, focusing on the dual goals of oncological safety and enhanced esthetic outcomes. The findings of this review, derived from 64 studies, highlight the critical aspects of the BRM, encompassing skin incision patterns, implant coverage strategies, nipple–areolar complex (NAC) strategies, and the choice between direct-to-implant (DTI) and two-stage reconstruction techniques.

The review confirms the versatility of the Wise pattern incision, which was the most commonly reported (34 studies). This incision effectively manages both vertical and horizontal excesses in macromastia and ptotic breasts. However, it is associated with notable complications, including skin necrosis (up to 27%) and delayed wound closure, especially at the T-junction. Modified versions of the Wise pattern have been proposed to mitigate these risks, emphasizing reduced flap length and tension at closure [29,30,31,32,33,34,35]. While alternative patterns such as the vertical, Passot, and batwing incisions demonstrate lower complication rates, their reshaping potential remains limited, particularly in cases of severe ptosis or macromastia. These findings suggest that incision selection should be guided by a balance between esthetic goals and patient-specific risk factors, such as BMI, smoking status, and prior radiotherapy.

Implant coverage strategies also play a pivotal role in BRM outcomes. The musculodermal pocket, created with an inferiorly based adipodermal flap (IWADF) [29], was the most frequently reported approach (37 studies). This method offers complete vascularized coverage, reducing implant exposure risk and supporting mastectomy flap healing. Despite its advantages, IWADF harvesting requires specific anatomical conditions, such as adequate nipple-to-inframammary fold distance, limiting its applicability in certain patients. The use of acellular dermal matrices (ADMs) and synthetic meshes provides alternative coverage strategies, particularly in prepectoral implant placement [47,48,49,50,51,52]. However, cost-effectiveness and complication rates associated with ADM use require further investigation [53,54,55,56,57].

NAC management strategies remain a critical esthetic consideration for the BRM. While some authors advocate for auto-free nipple grafting or delayed NAC reconstruction, immediate preservation on a dermal flap offers superior esthetic outcomes when it is oncologically safe [48,49,50,51,52]. The choice of NAC strategy must involve the consideration of factors such as required lift distance and mastectomy flap thickness to balance oncological safety and esthetic outcomes.

Finally, the choice between DTI and two-stage reconstruction techniques must be tailored to individual patient characteristics. Although DTI is associated with shorter recovery times and fewer surgical procedures, it carries a higher risk of complications, such as skin flap necrosis (9.7% vs. 4.7% in two-stage reconstructions). This highlights the importance of thorough intraoperative flap assessment to guide decision making and minimize risks [69,70,71,72,73].

This study is limited by the heterogeneity of the reviewed literature, which includes various study designs and inconsistent reporting of outcomes. Furthermore, the lack of standardized definitions and criteria for complications and esthetic outcomes limits the comparability of studies. Lastly, the absence of meta-analytic methods precludes quantitative synthesis of the data, highlighting the need for prospective, standardized research to further validate the findings and refine BRM techniques.

## 5. Conclusions

Breast reshaping mastectomy (BRM) introduces a novel paradigm in reconstructive breast surgery, merging oncological safety with the esthetic imperative of achieving a natural and harmonious breast shape. This review emphasizes that the choice of surgical techniques in the BRM—including skin incision patterns, implant coverage strategies, NAC preservation, and reconstruction timing—must be carefully tailored to individual patient needs and risk profiles.

Among the various incision techniques, the Wise pattern incision stands out for its reshaping potential but also presents significant risks that require meticulous intraoperative management. The musculodermal pocket emerged as the most effective implant coverage strategy, providing robust vascular support and reducing complications, though its applicability may be limited in certain anatomical scenarios. The management of the NAC—whether through immediate preservation, grafting, or delayed reconstruction—remains pivotal in determining esthetic outcomes.

Despite advancements, this review highlights persistent challenges, including the lack of standardized guidelines and variability in complication rates across techniques. Future research should focus on prospective studies and standardized protocols to refine BRM practices and improve patient outcomes. The BRM offers significant promise as a comprehensive approach to addressing both functional and esthetic goals in breast reconstruction, making it a valuable addition to reconstructive surgeons’ repertoire.

## Figures and Tables

**Table 1:** Characteristics of skin incision type in breast reshaping mastectomy. The symbol “+” indicates the capacity of each parameter, where “+++” represents an optimal capacity, “++” a good capacity, “+” a moderate capacity.

Incision Type	Reshaping Potential	Adipodermal Flap Creation	Mastectomy Skin Flap Vascularity
Standard Wise pattern	+++	+++	+
Modified Wise pattern	+++	+++	++
Vertical pattern	++	+	++
Passot pattern	++	++	++
Batwing pattern	++	++	++
Periareolar pattern	+	+	+++
Raquet pattern	+	+	+++
Modified B mammaplasty pattern	++	+	++

**Table 2:** Surgical tricks in breast reshaping mastectomy skin incisions.

Author	Surgical Trick
Querci della Rovere et al. [10]	A little triangle of epidermis can be preserved at the mid-clavicular level of the IMF to reduce tension at T-inverted skin closure.
Losken et al. [34]	Incision for the mastectomy inside the skin-reducing incision pattern to reduce stress on the final skin flap edges.
	Preserve the dermal triangle between the vertical incisions of the Wise pattern or modified Wise pattern as a barrier dermal flap to support the healing of the T-inverted skin closure.
Ross et al. [35]	The length of the vertical incision in the modified Wise pattern should be tailored on the nipple to IMF distance. Where there is >15 cm from the nipple to IMF, an 8 cm distance from the top of the V to the horizontal limb is marked. Where there is 20 cm or more from the nipple to IMF, this distance can be increased to 10 cm.
Dietz et al. [31]	Preserve 1–2 cm strip of deepithelialized skin along the superior flap for a healthier flap.
Ladzinski and Vlajcic [64]	The medial and lateral end points of the inframammary incisions are marked along the projection of the new inframammary fold, and care should be taken to keep them as short as possible using an M-plasty at the end points.

**Table 3:** Classification of implant coverage strategy.

Prepectoral		Subpectoral	
Non-ADF/ADM-Based	ADF/ADM-Based	Non-ADF/ADM-Based	ADF/ADM-Based
Subcutaneous	Partial coverage with ADF	Complete muscular/musculo-fascial coverage	Complete coverage with musculodermal pocket (composed by pectoralis major muscle superiorly and ADF inferiorly)
(with/without barrier dermal flaps)			
	Complete coverage with ADM + ADF overlay		Complete coverage with muscolodermal pocket + ADM lateral/underlying ADF/interposed between muscle and ADF or synthetic mesh underlying ADF
	Complete coverage with ADF + ADM/dermal patch or synthetic mesh		Complete coverage with muscle superiorly and ADM inferiorly

## Data Availability

No new data were generated in this study, except for the ones presented. No additional data are available.
